# Mapping and engaging the Costa Rican scientific diaspora: experiences from over a decade implementing the hipatia.cr portal

**DOI:** 10.3389/frma.2026.1856801

**Published:** 2026-07-17

**Authors:** Esteban Duran-Monge, María Santos

**Affiliations:** 1 Programa Estado de la Nación, San José, Costa Rica; 2 Independent consultant, San José, Costa Rica

**Keywords:** brain linkage, brain-drain, Costa Rica, global south, international mobility, migration, scientific diasporas, skilled

## Abstract

By describing the framework developed by the hipatia. cr initiative for mapping the Costa Rican scientific diaspora and engaging it with local actors and sectors, this policy and practice review provides a practical guideline that may be useful for other emerging countries seeking to connect this valuable resource to their country of origin. This initiative was launched in 2012 by the State of the Nation Program of the National Council of Rectors, to provide a yearly updated overview of the country's STI capabilities and supporting public-private decision-making processes. Implementing the Costa Rican scientific diaspora framework has involved five sequential steps: a) Gather initial contact information from various sources to establish the preliminary pool of potential members; b) Design and implementation of an online data-collection form aimed at identifying, mapping, and characterizing this scientific community abroad; c) Develop a database and an online dashboard integrated into the hipatia.cr portal, to make relevant information about the profile of this community publicly accessible; d) Introduce and position the topic within the national narrative and mobilizing diverse actors such as academic, public institutions, policymakers, media, and industry sectors; e) Design innovative mechanisms to engage them with diverse local stakeholders, as well as supporting monitoring in national STI policies. The resulting mapping currently constitutes the most comprehensive registry of the Costa Rican scientific diaspora available in the country. Several aspects of this initiative intersect with key development goals and main challenges faced by the country.

## 1 Introduction

Science, Technology, and Innovation (STI) are main drivers of general wellbeing for countries, and are essential for socio- economic progress and progressive transformation of societies. Given that Costa Rica is a country with a limited territorial extension, that lacks large-scale monoculture areas or strategic raw materials such as oil, and whose main source of wealth—biodiversity—can only be used under the condition that it is not destroyed, the need for an economy grounded in STI becomes even more critical. This is further reinforced in the country's structural changes in the productive system over recent decades, which have increased the strategic importance of knowledge-intensive sectors including advanced manufacturing, information and communication technologies, and technology-based firms. Nevertheless, the limited availability of highly skilled talent and the difficulty of addressing these shortages in the short term (PEN, 2025) remain among the main challenges to expand these dynamic sectors and to harness the potential of STI for sustainable development.

This challenge has been highlighted by other organizations, such as the Organization for Economic Co-operation and Development (OECD), which identified the mismatch between graduates' skills and the needs of industry and academia as one of the key weaknesses of the national innovation system^1^ (OECD, 2017), a matter that exacerbates international gaps in productivity and income. In this context, the scientific diaspora represents a highly valuable asset that Costa Rica cannot afford to overlook.

In this article members of the Costa Rican scientific diaspora (CRSD) are considered people who recognize themselves as Costa Ricans and possess graduate or postgraduate studies in the areas of engineering, medical, natural or agricultural sciences, and who are currently living abroad, whether for study or employment reasons. It is indeed a dynamic community, as its members may leave or return over time.

Beyond considerations of whether they return or not, or of framing this phenomenon as “brain drain,” initiatives related to the CRSD are better situated within a different paradigm that focuses on connecting them to their country of origin. Even if they stay abroad, they can still be of significant benefit to their home countries, provided that these connections are maintained, as they can make important contributions through ideas, experiences, collaborative projects, professional networks, or remote work in support of national development.

In Costa Rica, the first initiative to identify members of the CRSD was the creation of the TICOTAL Network^2^ in 2010, an instrument developed by the National Academy of Sciences of Costa Rica (ANC, 2026), which currently contains academic information on approximately 601 individuals.^3^ In addition, this program gathers members' perspectives on their experiences abroad, the preconditions for returning home, and recommendations for advancing STI in Costa Rica, among other relevant issues, as consolidated by Jarquin-Solis et al. (2022). Furthermore, TICOTAL aims to disseminate the country's scientific and technological activities internationally.

Building on this initial effort, the State of the Nation Program (PEN) of the National Council of Rectors and its hipatia.cr portal, in relation to the CRSD, proposed a broader strategy whose initial objectives were to map and characterize the specific features of this community and subsequently make this information publicly available. As these efforts progressed, various mechanisms were designed to connect this talent with different local sectors, some of which have been previously described by (Santos-Pasamontes, 2022). This paper presents this framework and its main results, and in the context of a Policy and Practice review, it should be read as a practical guideline that may be useful for other emerging countries seeking to map and connect this valuable resource with their countries of origin.

The hipatia.cr initiative aligns with other efforts aimed at mapping, studying, and engaging scientific diasporas across Central and Latin America. Over recent years, various initiatives have sought to study these communities, identify highly skilled nationals abroad, better understand their profiles and trajectories, and promote their linkage with domestic scientific and productive systems. In Costa Rica, (Jarquin-Solis et al. 2022) analyzes 10 years of 121 interviews from TICOTAL and characterizes fields, destinations, funding, current locations, engagement with Costa Rican STI, and policy lessons.

Other works give a Central America comparative perspective. (Bonilla et al., 2022b) present a review of Converciencia as a practice to engage the Guatemalan scientific diaspora. It also offers a regional review of policy instruments, including: Mexico's Red Global MX, Brazil's Rede Diáspora Brasil, Uruguay's CUAC, and Costa Rica's hipatia.cr as formalized engagement mechanisms. Another work from (Bonilla et al., 2022c) presents a qualitative study based on focus groups and interviews on Guatemalan scientific diaspora, and discusses policy gaps and potential of digital/AI-based mapping. (Alvarado et al., 2022) present a case study on the development of a digital technology system called ALMA aimed to address COVID-19 health needs in Guatemala, that illustrates how organized scientific diasporas contribute via remote, flexible, tech-enabled engagement.

In Honduras, Bonilla et al. (2022a) analyze three initiatives—Honduras Global, Organization for Women in Science for the Developing World (OWSD), and Asociación de Graduados de la Escuela Agrícola Panamericana- Zamorano (AGEAP)—highlighting the absence of official registries and framing the “brain drain” perspective as an opportunity for “brain circulation.” Similarly, Bonilla et al. (2021) document the scarcity of directories for women scientists and the Central American diaspora, emphasizing the role of informal social media networking and the urgency of including women in mapping and community-building efforts. Regarding other regional studies, Gómez-Flores et al. (2022) interviewed 29 Mexican scientists in the US and Canada to identify factors that enable or obstruct collaboration. Finally, Avendano-Uribe et al. (2022) present the Science Clubs Colombia case, demonstrating how the diaspora can be mobilized for Science, Technology, Engineering, Arts, and Mathematics (STEAM) workshops in underserved communities through scalable models of educational engagement.

With a broader scope, (Echeverría-King et al., 2022) provides a regional map of organized scientific-diaspora networks and lists hipatia.cr as a Costa Rican global network, alongside other initiatives in Latin America and the Caribbean. A more recent work (Echeverría-King et al., 2025) offers a comparative roadmap of strategies to transform brain drain into knowledge circulation and position scientific diasporas as “permanent partners” in innovation and policymaking across Latin America and beyond, including: Argentina-RAICES Program; Mexico-Red Global MX; Colombia-Red Caldas; Costa Rica-hipatia.cr; Guatemala-Converciencia and RedCTI; Uruguay-CUAC Program; and Chile-PAI Programs and CONICYT Return Program.

This article aimsto describe and disseminate a comprehensive empirical framework, organically refined over more than a decade, designed to map and characterize the CRSD. It examines its strategic engagement with diverse national sectors and the integration of this topic into the public agenda, with the ultimate objective of leveraging scientific human capital for national development. Furthermore, the paper provides the underlying methodology, along with practical tools and evidence-based recommendations.

## 2 Methods

The framework aims to provide a systematic approach for mapping and engaging the CRSD. As described in the next section, the whole process comprises five interconnected stages (see Table 1).

**Table 1:** Interconnected stages comprised by the framework

Stages	Description
1	Establishing a preliminary pool of potential members of the CRSD
2	Developing and deploying an online data-collection instrument to identify, map, and characterize diaspora members
3	Developing a profiling database and an online dashboard
4	Profiling the CRSD and positioning the topic within the national STI agenda and public narrative
5	Designing and implementing engagement mechanisms involving diaspora members and local stakeholders

The main sources for the proposed framework are databases or listings of potential scientific diaspora members gathered from several institutions, as well as social network searches and contacts provided directly by other individuals of the diaspora and STI professionals in the country (for the specifics see section 3).

The study was initiated in 2012 and is implemented as an ongoing process reproduced on a yearly basis. In 2014 a first version of the mapping and characterization of the CRSD was published in Costa Rica's State of Science, Technology and Innovation Report (PEN, 2014). Afterwards, in 2015 the analysis was both upgraded and updated, and published as a web app as part of the first version of the hipatia.cr portal.

Since then, this process has been conducted annually, covering a 12-year period from 2014 to 2025. Throughout this period, the methodology, data sources, and analytical procedures were progressively refined and expanded. These iterative improvements ultimately led to the development of a comprehensive framework, which is described systematically for the first time in this paper. As result, the first implementation of the framework, conducted in 2014, combined an initial mapping exercise with the participation of 222 individuals. Through successive annual updates, the initiative expanded its coverage, reaching a total of 834 diaspora members by 2025.

## 3 A framework for mapping and engaging the Costa Rican scientific diaspora

Developed and refined over more than a decade, the framework combines multiple data sources, digital tools, analytical procedures and engagement mechanisms to generate evidence on the scientific diaspora and strengthen its connections with the national STI ecosystem. Together, the stages described in this section, transform dispersed information into actionable knowledge.

### 3.1 Stage 1. Establishing a preliminary pool of the potential members of the CRSD

The framework was launched in response to a fundamental challenge: the notion that a valuable, growing community of highly skilled Costa Rican scientists was residing abroad, coupled with the absence of a comprehensive database to characterize them. Accordingly, the first step focuses on identifying members of the CRSD, drawing on multiple sources:

- Members of the established TICOTAL network. This network served as a foundation for developing the initial version of the preliminary pool.
- Scholarship holders. Data bases of scholarship holders from public universities and other organizations, like CONICIT^4^ and CR-USA.
- Costa Ricans working abroad according to social networks. Systematic searches made through Linkedin, using search strings to identify graduates from national universities located in foreign countries. After identifying, individuals were contacted and invited to participate in the study.
- Contacts of local professionals. Contact information facilitated by local professionals related to the STI sector in the country. This information expanded the initial pool build from TICOTAL.
- Contacts of CRSD members. Additional contacts shared by CRSD members that participated in previous versions of this study.

As a result, an ongoing database was developed with contact information—hereafter referred to as contact database—which has been updated and consequently growing on a yearly basis. As per 2026, it integrates contact information of 889 members and potential prospects of the CRSD participating in the study.^5^

### 3.2 Stage 2. Development and deployment of an online data-collection form to identify, map, and characterize the CRSD

To collect data, an open online questionnaire was developed with the LimeSurvey Tool for the overall characterization of the CRSD. The initial version was based on the form from Tejada et al (2013) developed for the project “Connecting the scientific diaspora of the Republic of Moldova to the scientific and economic development of the home country.” ^6^ Subsequent versions were refined based on the needs and feedback of local stakeholders. The last version of the questionnaire is available online permanently.^7^

The form consists of 33 questions, which are organized in five sections:

- General profile.
- Work and study situation.
- Linkages and current connections with local STI communities.
- Suggested cooperation mechanisms to collaborate with local communities.
- Drivers of migration and intentions to return to Costa Rica within the next 5 years.

Individuals in the contact database are invited to complete the online form. Due to the constant evolving nature of this community, the consultation process—including the form—is deployed annually to update data and expand our reach.^8^ Following the initial consultation, two versions of the form were launched: one for new potential members and another for existing members to update their information and confirm their residence abroad. Historical response rates have not been systematically recorded however, in 2025 the response rate reached 18% of the individuals invited to participate in the online consultation.

### 3.3 Stage 3. Development of the profiling database and an online dashboard

Next steps include the development of the profiling database and online dashboard available at hipatia.cr. This ensures that relevant information regarding the CRSD participants in the study is publicly accessible. The profiling database is generated when contacted CRSDs (individuals in our contact database) complete the online questionnaire. The steps of this stage are the following:

- The “raw” data captured through the online form starts an ETL (extract, transform and load) process using Pentaho Business Intelligence and Data Integration platform on its Community Edition. First, data is extracted from the LimeSurvey response table. Afterwards, it is carefully reviewed and enters a transformation pipeline to create a usable and trusted resource. The pipeline includes several steps. Duplicated individuals are identified and removed based on name, email and location fields. A unique ID is assigned to each person. Data cleaning is performed over some of the variables, for example information regarding location and city of residence. Science and technology fields classification is applied based on the Frascati Manual 2015 (OECD, 2015) guidelines for measurement of scientific, technological and innovation activities. Lastly, the system excludes responses from individuals who reported having already returned to the country or planning to return within the next 2 months, as well as incomplete responses.
- At the end of this process, data is normalized and loaded on the MySQL open-source relational database management system. The result is a curated version of the CRSD “profiling database,” that comprises 43 variables distributed in 3 data tables, ready to enter the analytics phase. As of 2025, a total of 834 individuals are included in the profiling data base. That distribution of the respondents by source for 2025 show a major representation of scholarship holders (32%), followed by contacts directly facilitated by diaspora members and local contacts (28%), Linkedin (24%) and the TICOTAL network (16%)
- The data is then structured into cubes using Online Analytical Processing (OLAP) technology through the Pentaho Saiku Analytics component. This tool allows exploration and analysis tasks via intuitive drag and drop interface. Based on these tasks, a refining process is conducted, to prioritize and select the information that will be incorporated in the final data visualizations. For instance, the questionnaire includes 33 questions across all the modules, from this full set a selection of 15 key variables is retained. All information is presented in aggregated form and never at the individual level, in order to ensure the protection of personal data and safeguard respondents' privacy.
- The final queries from the OLAP cubes are used for the development of the online dashboard using Pentaho data viz components. At this point, the selected variables and their relationships are translated into interactive visual components that allow users to navigate the data effectively. The dashboard incorporates export functionalities and drill-down capabilities, enabling users to move from aggregated views to more detailed levels of information and to extract data for further analysis.
- A data validation protocol is then applied to ensure accuracy and reliability. This process includes verifying consistency between source data, the profiling database, OLAP cubes, online data visualizations and export outputs, as well as checking the correctness of aggregations, cross-tabulations and indicators. This step guarantees that the dashboard faithfully represents the curated dataset and supports robust analytical use.
- The reviewed version of the dashboard is then published in the Hipatia portal, that allows users to explore and export the data.^9^

### 3.4 Stage 4. Profiling the CRSD and positioning the topic in the national narrative

**Figure 1:**
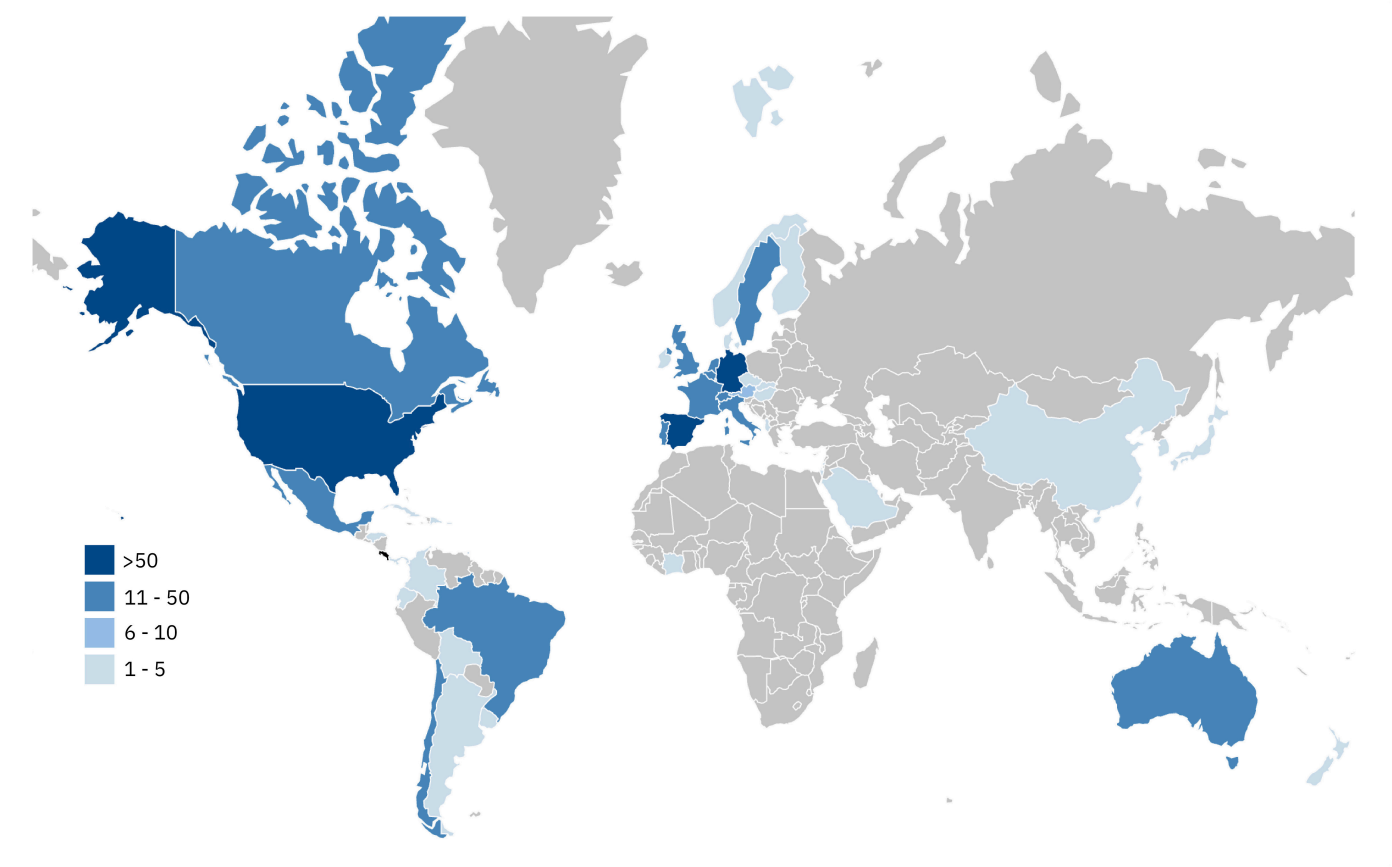
Geographical distribution of members of the scientific diaspora participating in the study by country (number of individuals).

Once the previous stage was completed and a database suitable for analysis was established, the community profile reveals its high strategic value for the country. Although not of great size, it unites a group of professionals with a formidable academic preparation. The latest results show that 81% of respondents to the 2025 questionnaire hold postgraduate degrees. Members are distributed across more than 47 countries, with a strong concentration in countries at the forefront of global scientific and technological development, particularly the United States and Germany (Figure 1). They are trained across a broad range of STEM fields, showing complementarity with areas where local talent shortages are most pronounced. Notably, 36% hold postgraduate degrees in fields where the country faces the greatest gaps, such as Information and Communication Technologies (10%) and Electrical and Electronic Engineering (5%), disciplines in which domestic supply does not meet demand.

Given its strategic value, efforts to position this thematic among the national discussion were made from multiple perspectives, with the aim of integrating it into both public and private decision-making processes. The displayed approach builds upon highlighting the academic background of the CRSD, but additionally implemented a more propositive approach, that considers the design of innovative mechanisms to engage them with diverse local stakeholders, as well as supporting monitoring in national STI policies and the formulation of potential policies aimed to facilitate their reintegration (see stage 5).

To position the topic, constant joint efforts of the hipatia.cr researchers and State of the Nation Program diffusion team has been made. Since the launch of the portal in 2015, a total of 2019 STI pieces have been produced and disseminated in Costa Rican media, of which 28 are directly associated with the CRSD. Blogs, interactive data visualizations, infographics and videos are among the used formats. Some examples are:

- Infographic: “Why I am not returning to Costa Rica?” It promotes reinsertion of CRSD individuals by pointing out a series of conditions and incentives required for return, as indicated directly from the community members. This underscores challenges that the country must attend, not only for diaspora member reintegration, but also for the improvement of the national STI ecosystem. It is available in the following link: https://hipatia.cr/aportes/infografia-por-que-no-regreso-costa-rica
- Video: “Costa Rica needs more women in science and technology careers”. Aimed at promoting scientific vocations in the country's young women. It was made in collaboration with members of the diaspora who are regarded as role models due to their outstanding professional performance. It is available in the following link: https://hipatia.cr/video-costa-rica-necesita-mas-mujeres-en-carreras-de-ciencia-y-tecnologia
- Data visualization: “Profiling the Costa Rican Scientific Diaspora: A Valuable Asset for National Engagement.” Interactive visualization based on the CRSD dataset and developed with the Tableau Public tool. Introduces storytelling to address the general profile, gender gap, return plans and the importance of connecting this asset with the country's STI ecosystem. It is available in the following link: https://hipatia.cr/aportes/radiografia-de-la-diaspora-cientifica-costarricense-un-valioso-activo-para-vincular-con-el~

### 3.5 Stage 5. Design engaging mechanisms with local stakeholders

PEN has designed and implemented a variety of mechanisms to engage the CRSD with local stakeholders. Some of them were based on feedback or direct requests by local actors. Some important mentions are:

- Supporting the monitoring of two national STI policies through the generation of indicators included in national plans of the Ministry of Science, Innovation, Technology and Telecommunications: the National Science, Technology, and Innovation Plan 2022–2027 and the National Policy on a Knowledge-Based Society and Economy 2022–2050.
- Engaging members of the CRSD for multiple purposes: addressing job and research vacancies in universities and private firms that local professionals cannot fill; evaluating projects and acting as technical mentors in workshops; and leveraging their professional contacts to aid national organizations in attracting foreign direct investment. The latter exemplifies the diaspora networking actions described by Tejada et al. (2013), which are based on the logic of connectivity, the multiplier effect of personal ties to the home country, and their subsequent role as development actors.
  In thesecases, hipatia.cr acts as a communication channel between interested organizations and individuals registered in the CRSD profiling database, facilitating matchmaking between both parties when certain criteria are met. Once a first contact is made, the organizations are in charge of the matchmaking management and formalization on any possible vinculation process.
- Integration of diaspora members in public programs evaluation committees: the Development Banking System of Costa Rica drew on the HIPATIA database to identify experts who could serve as consultants on the Evaluation Committee of the Pilot Venture Capital Program in 2019. This information made it possible to incorporate specialized criteria in the selection of candidates for capital funding, thereby strengthening the transparency and technical quality of the process.
- Development of an *ad-hoc* platform to facilitate the participation of more than 100 experts -mainly in health and molecular biology fields—as mentors at the HackCovid19CR Hackathon held during the Covid pandemic. The tool built for this purpose is still publicly available at: https://hipatia.cr/la-plataforma-hipatia-del-programa-estado-de-la-nacion-colabora-en-el-abordaje-de-las-necesidades.
- Development of an online platform to enable the participation of the CRSD as technical mentors of local startups through a mentorship program created by the hipatia.cr initiative. At the date of preparation of this paper, this tool is not available, as it is undergoing a redesign.

It is worth mentioning that the latter two platforms aforementioned were designed in a form that would enable the necessary conveyance of technical information while ensuring the protection of the individuals contact information.

**Figure 2:**
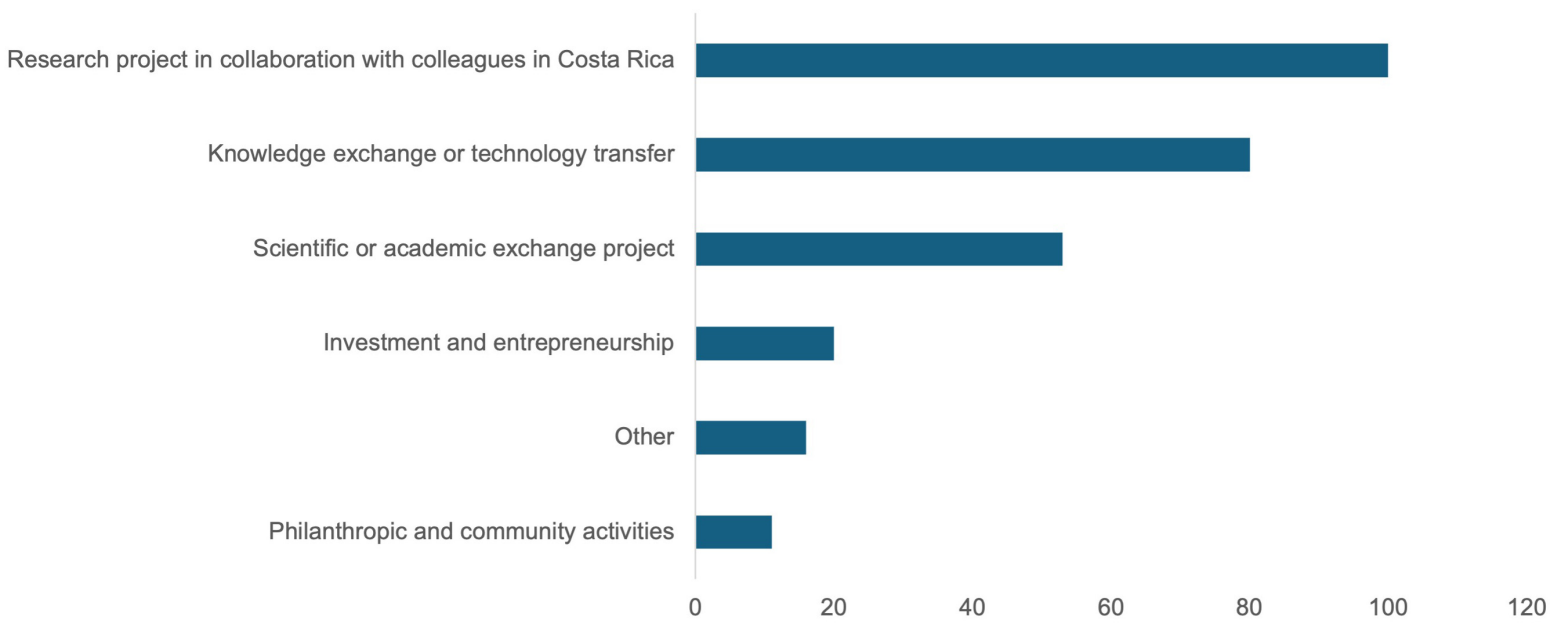
Cooperation initiatives with the country undertaken by members of the CRSD that participated in the study. 2025 (number of mentions).

Furthermore, the collected data made it possible to identify the cooperation initiatives with Costa Rica undertaken by members of the CRSD that participated in the study. CRSD's current local community engagement initiatives prioritize academic activities such as joint research, as well as the transfer of scientific and technological knowledge (Figure 2). A total of 280 actions were mapped. Although it cannot be stated that these specific initiatives are a direct result of the framework's implementation, they provide an indicator of the types of actions undertaken by the members of this community, some of them during the years in which the framework has been in operation. As future work, specific indicators could be developed to assess the framework's contribution to fostering collaborative initiatives.

## 4 Discussion and actionable recommendations

Costa Rica lacks a cohesive strategy to attract highly skilled professionals who can contribute to advancing science, technology and productivity. Although the government seems to have recognized the strategic importance of its scientific diaspora and has incorporated this into policy plans, a practical follow-up initiative has not yet been implemented, and only recently a new policy is being promoted to fill this gap. Consequently, the authors urge the development of a long-term program designed to formalize a permanent diaspora engagement policy. This initiative should focus on two simultaneous fronts: creating the necessary conditions to facilitate return and reintegration, while strengthening engagement mechanisms for those who choose to remain abroad.^10^

Each of these approaches could build upon the mapping, characterization, and networking initiatives developed by the PEN. Furthermore, they should align with the potential complementarity between the CRSD and local needs and opportunities, particularly in areas where national talent scarcity (PEN, 2025) intersects with the country's core challenges. Understanding the underlying reasons behind the decision to return—or not—can inform the design of effective repatriation policies.

Regarding the engagement of those planning to stay abroad, the authors recommend creating a program endowed with human and financial resources to establish a networking platform. This platform would promote connections among CRSD members, as well as with scientists, NGOs, diplomats, civil society, government, and industry within the country. Such a platform could be organized into chapters based on the diaspora's location, interests, and affiliations. Finally, future lines of action to be explored by hipatia.cr include gathering data not only on the type of actual engagements, as it is already registered, but on the specific activities involved -such as the number of manuscripts or research proposals originating from collaborations, number of students trained, participation on decision making-. These indicators could be converted into a more formal monitoring framework. There is also opportunity of conducting analytical studies to better understand the influence of their main activities (students, scientists, researchers, and professionals) in host countries, alongside factors influencing their return plans, and the nature and scope of potential collaborations with Costa Rica.

Regional efforts in diaspora engagement show that academic collaborations are the most frequent form of interaction with scientific diasporas (Gómez-Flores et al., 2022; Piñeros Ayala et al., 2025). Within this context, initiatives have focused on identifying collaboration opportunities between the diaspora and research groups, as well as fostering student mobility programs (Jarquin-Solis, 2022). This aligns with the nature of current engaging initiatives undertaken by CRSD members (Figure 2).

In this regard, the main obstacles to successful collaborations faced by some Latin American countries in engaging with their diaspora are also worth considering. These include the lack of knowledge regarding available mechanisms, a mismatch of timelines between academic and non-academic activities, and difficulties in setting common goals and objectives. Findings indicate that collaborations between academic organizations—compared to NGOs or governments—are easier due to the shared aim of conducting research. Conversely, in the case of the latter, the main challenges have been establishing common goals and schedules due to the differing timeframes of NGOs and government institutions (Gómez-Flores et al., 2022; Piñeros Ayala et al., 2025).

On the other hand, potential policies within a program aimed at subsidizing the return and reintegration of diaspora members, as well as attracting foreign migrants, are diverse and extend beyond economic and labor-related incentives. When consulted about the conditions that would facilitate their return to Costa Rica, CRSD members emphasized public and academic policy factors, general institutional working conditions—i.e., time potentially allowed to research as opposed to mainly teaching-, as well as infrastructure and equipment for undertaking research and development (R&D), along with incentives for professional development and global connectivity. These also include, among other aspects, the importance of an environment free from bureaucratic or legal barriers, that is open to intersectoral and global interaction (PEN, 2026).

The OECD has recommended to their member countries the adoption of policies to promote the permanence and attraction of specialized talent and propose a methodology that considers more than 50 indicators to develop regional attractiveness profiles covering six domains of attractiveness: economic attraction, connectedness, visitor appeal, natural environment, resident wellbeing and land-use and housing (Andersson 2025). Key lessons learnt from OECD Indicators of Talent Attractiveness 2019 (ITA) show that country's attractiveness can be high for certain types of talented migrants even as it is lower for other potential migrant categories, i.e., highly skilled workers at master/PhD level, international students in tertiary education, foreign entrepreneurs and start-up founders (OECD, 2019). Costa Rica, along with other countries like Türkiye, Colombia, Mexico, Greece and Israel consistently rank at the lower end for all migrants profiles at OECD indicators of talent attractiveness 2023 rankings along the different dimensions and variables to the aggregated ITA scores^11^ (Andersson, 2025).

At least 24 OECD member countries offer tax relief programmes to attract highly skilled foreign migrants and returning nationals, including reduced income tax, flat rates, and exemptions for foreign-sourced income (OECD, 2024). In Costa Rica, financial incentives originally included in the Law for the Promotion of Science and Technology Development N° 7169^12^ of 1990, were repealed. Other incentives, proposed later on as an *ad hoc* component of a loan for the country's government obtained in 2014 from the Inter American Development Bank, directed to companies developing R&D projects, were not used by the sector.

Given the local scarcity of highly skilled individuals, since the profile of the CRSD has proven to be an important asset, its effective engagement entails a critical factor for the country's development as they could help address existing skills gaps and foster competitiveness, productivity, and technological development.

As demonstrated previously, the annually updated database developed by PEN can serve as a vital link between the CRSD and local industries to implement this initiative. Concurrently, these efforts should be complemented by academic initiatives aimed at engaging expatriate members with local research communities to strengthen scientific development, as exemplified by the TICOTAL Network (ANC, 2026).

### 4.1 Challenges, lessons learned, and contributions of the hipatia.cr framework

The policy and practice experience presented in this paper offers a conceptual and empirical framework that may be useful for other emerging economies in effectively leveraging this valuable resource to their country of origin. Furthermore, this section synthesizes the key challenges, lessons learned and contributions derived from over a decade of the design and implementation of this framework.

The authors identify two primary challenges. The first is a major bottleneck caused by the lack of an exhaustive national registry of the CRSD, for which the TICOTAL Network served as the closest available source. Other sources, such as Linkedin and data bases of scholarship holders from several organizations, entail significant limitations regarding data selection, meaning some of the members of the CRSD may never be identified and the resulting data does not represent a statistically significant sample. Despite this limitation, the databases developed by PEN over the years constitute the most comprehensive registry in the country to date. As noted in Stage 1, using multiple sources has been crucial to addressing this gap.

Once the contact and profiling databases are established, the second challenge lies in maintaining accuracy while tracking such a dynamic community. This involves two key aspects: first, expanding coverage—as new scientists continuously join the diaspora, requiring ongoing efforts to identify and incorporate them—and second, ensuring data accuracy by removing individuals who have returned to the country.

Similarly, the involvement of other public and private organizations has proven essential to sustainably expand database coverage, offering a key lesson in addressing the aforementioned challenges. Furthermore, the authors recognize that anchoring the project within prestigious and legitimate institutions is a determining factor for successful implementation. In this case, this was achieved through the participation of the State of the Nation Program (showcased on hipatia.cr) and the National Academy of Sciences of Costa Rica, through the preceding TICOTAL Network.

Finally, through extensive institutional experience, a practical five-stage framework was implemented to transform raw information into evidence, tools, and engagement mechanisms. This simultaneously fostered stakeholder involvement and advanced the topic within the national discourse. Overall, the data, outputs, outcomes, and broader policy effects detailed below have served as a critical foundation for amplifying reach and impact:

- **Source data identification:** this provides the basis for identifying diaspora members and includes databases of potential scientific diaspora, social network data, and individual contacts provided by STI professionals and other diaspora members. [1]
- **Outputs:** these consist of a consultation instrument (specifically an online data-collection form), the CRSD database, the profiling of the CRSD, and a mechanism for public data accessibility dashboard, in aggregated format, ensuring personal data protection and respondent privacy. The latter was implemented here via an interactive dashboard available at the hipatia portal. Other outputs consists of interactive data visualizations, and tailored actions addressing the needs of various stakeholders.
- **Immediate outcomes:** increased visibility of the topic, dissemination of information, and stakeholder engagement via the designed mechanisms.
- **Intermediate policy effects:** evidenced by the incorporation and use of diaspora-related indicators in national STI policy processes.
- **Broader development impacts:** the attraction of talent and increased collaboration with local communities.

These initiatives can be categorized into three distinct levels:

- **Level 1: Strategic positioning**. This involved the continuous production of diverse content—ranging from interactive visualizations to media pieces—to promote a sustained communication strategy. This effort has resulted in roughly 30 publications in national media since 2015.
- **Level 2: Engagement and reintegration initiatives**. In alignment with broader diaspora engagement approaches, the framework implemented a dual-action strategy designed to support both returning and non-returning stakeholders, as outlined in Stage 5.
- **Level 3: Policy input generation**. The sustained development of the aforementioned actions (Levels 1 and 2) enabled a higher level of impact, evidenced by the integration of diaspora indicators into current national science and technology instruments.

## Footnotes

1 In addition to weak government support and funding, as well as institutional fragmentation (OECD, 2017).

2 It can be accessed via the following link: http://ticotal.cr/conozca-acerca-de-ticotal.html.

3 As of April 2026.

4 Later this organization was renamed as Promotora Costarricense de Investigación e Innovación.

5 In 2026, a comprehensive database-cleaning process was conducted to remove outdated contacts who had never responded to the questionnaire. As a result, the contact database was reduced to 889 members. Consequently, the number of contacts reported in this study differs from that used in previous years, which may explain the apparent discrepancy between the response rate reported below and the number of individuals included in the profiling database (see Section 3.3).

6 Conducted by the École Polytechnique Fédérale de Lausanne (EPFL) and the Academy of Sciences of Moldova (ASM), and funded by the SCOPES Programme of the Swiss National Science Foundation (SNF). The original form was contributed by and edited with the permission of Gabriela Tejada, a scientist at the Cooperation and Development Center at the Ecole Polytechnique Fédérale de Lausanne, where she is Project Leader of research on scientific diasporas and skilled migration.

7 The latest version is available at: https://formmore.com/f/hipatia/registro-de-di-spora-cient-fica-costarricense-d2abcd.

8 Data actualization includes subtracting from “contact database” and “profiling database” the individuals indicating their return to Costa Rica.

9 Making it publicly available at this link: https://hipatia.cr/dashboard/diaspora-cientifica.

10 As per the last consultation implemented in 2025 to the professionals who responded the on-line interview, 42% of them are planning to return to Costa Rica in the next 5 years.

11 Dimensions considered: quality of opportunities, skills, environment, income and tax, inclusiveness, future prospects, quality of life and family environment OECD, 2025.

12 In its original version, Law 7169 on the Promotion of Scientific and Technological Development established an incentive regime that included tax deductions for researchers upon their return to Costa Rica; however, these provisions were subsequently almost entirely repealed.
